# Task requirements affect the neural correlates of consciousness

**DOI:** 10.1111/ejn.15820

**Published:** 2022-09-26

**Authors:** Lau M. Andersen, Mikkel C. Vinding, Kristian Sandberg, Morten Overgaard

**Affiliations:** ^1^ Center of Functionally Integrative Neuroscience Aarhus University Aarhus Denmark; ^2^ Aarhus Institute of Advanced Studies (AIAS) Aarhus University Aarhus Denmark; ^3^ Department of Linguistics, Cognitive Science and Semiotics Aarhus University Aarhus Denmark; ^4^ NatMEG, Department of Clinical Neuroscience Karolinska Institutet Stockholm Sweden; ^5^ Danish Research Centre for Magnetic Resonance, Centre for Functional and Diagnostic Imaging and Research Copenhagen University Hospital—Amager and Hvidovre Copenhagen Denmark

**Keywords:** consciousness, magnetoencephalography, perception, vision

## Abstract

In the search for the neural correlates of consciousness, it is often assumed that there is a stable set within the relevant sensory modality. Within the visual modality, the debate has centred upon whether frontal or occipital activations are the best predictors of perceptual awareness. Although not accepted by all as definitive evidence, no‐report and decoding studies have indicated that occipital activity is the most consistently correlated with perceptual awareness whereas frontal activity might be closely related to aspects of cognition typically related to reports. However, perception is rarely just passive perception *of* something, but more or less always perception *for* something. That is, the task at hand for the perceiver may influence what is being perceived. This suggests an alternative view: that consciousness is not one specific ‘function’ that can be localized consistently to one area or event‐related component and that the specific attributes of the neural correlates of consciousness depend on the task at hand. To investigate whether and how tasks may influence the neural correlates of consciousness, we here contrasted two tasks, a perceptual task and a conceptual task, using identical stimuli in both tasks. Using magnetoencephalography, we found that the perceptual task recruited more occipital resources than the conceptual task. Furthermore, we found that between the two conditions, the amount of frontal resources recruited differed between different gradations of perceptual awareness partly in an unexpected manner. These findings support a view of task affecting the neural correlates of consciousness.

## Graphical abstract

We here show that the neural correlates of consciousness cannot be disentangled from the task that humans are engaged in. We specifically show an interaction where the neural correlates of consciousness in frontal and occipital areas are affected differently depending on whether participants have to engage with the *perceptual* or the *conceptual* attributes of identical stimuli. These results indicate that to understand *perceptual consciousness* of individuals, one also has to understand the *action* and the *aim* of the task that individual is engaged in.

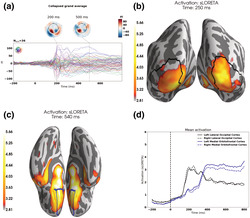

**Abbreviations**
ACE: almost clear experience
CE: clear experience
EEG: electroencephalography
LP: late positivity
MEG: magnetoencephalography
NE: no experience
PAS: Perceptual Awareness Scale
VAN: visual awareness negativity
WG: weak glimpse

## 1 INTRODUCTION

The search for the neural correlates of consciousness has been defined as the search for the minimal conditions sufficient for realizing a conscious representation (Chalmers, 2000). Using contrastive analyses, comparing otherwise identical stimulation that sometimes gives rise to a subjective experience and sometimes not (Baars, 1988; Crick & Koch, 1990), much research has been done isolating these minimal conditions sufficient for realizing a conscious representation. When studying visual perception, non‐invasive electrophysiological measures such as electroencephalography (EEG) and magnetoencephalography (MEG) reveal at least two candidates aspiring to be the ‘best’ neural correlate of consciousness (Förster et al., 2020), the early visual awareness negativity (VAN), in occipital and temporal areas, and the late positivity (LP), in parietal and frontal areas. However, relatively recent ‘no‐report’ studies (e.g., Frässle et al., 2014; Pitts et al., 2014) have provided strong evidence that the late frontal activity is related to the reporting of the conscious representation rather than the conscious representation itself (Förster et al., 2020; Koch et al., 2016), but see also Boly et al. (2017), Odegaard et al. (2017) and Michel and Morales (2020) for a debate of the results. This leaves the early visual activity as the main candidate for a neural correlate of consciousness, as also indicated by decoding studies showing that activity in visual cortex is more predictive of perceptual awareness than activity in frontal cortex (Andersen et al., 2016; Sandberg et al., 2013). In auditory awareness studies, similar results show that the early activity reflects awareness and the later frontal activity the task relevance (Schlossmacher et al., 2021), but see also the study of Eklund et al. (2019).

However, this debate assumes that there must be one particular neural correlate of consciousness. An alternative view argues that consciousness is not one specific ‘function’ that can be localized in this way and that the neural correlate of consciousness depends on the content of the experience (Mogensen & Overgaard, 2020). It is also becoming increasingly clear that perception is rarely just passive perception *of* something, but is almost invariably embedded in some context making it perception *for* something: Perception does not occur only as a passive process but is linked in a purposeful manner to action (Buzsáki, 2019). That is, depending on what action perception is *for*, one could expect that perception of otherwise identical stimuli will give rise to different patterns of neural activity, in other words reflecting the cognitive strategy (Mogensen & Overgaard, 2020) that is used to solve the task. Where to direct attention may be part of one's cognitive strategy. Studies of attentional manipulation have provided support for this view. For example, using positron emission tomography, Corbetta et al. (1991) showed that attention to different features of otherwise identical stimuli would elicit different patterns of activation in the visual cortex and beyond. Similarly, Kamitani and Tong (2005) showed that attentional focus on one of two overlapping, orthogonal gratings modulated visual cortex activity. It is important to note, however, that these earlier studies examined strong, overt attentional focus on one stimulus aspect and direct or indirect suppression of other aspects, and in at least the latter study, the visual experience varied dramatically between conditions. Consequently, if one is interested in knowing whether the task in itself changes the correlates of consciousness when attentional focus and visual experience are similar between tasks, a different approach is warranted. Our approach is to use a paradigm where participants need to attend the same visual features to solve a task that then varies in being conceptual or perceptual as explained below. We use a between‐subject design to further ensure that participants are not focused on the contrast between tasks and need to suppress attention to the alternative task. To make sure that identical stimuli would result in different levels of clarity of experience, we presented stimuli at low contrast using backward masking (Breitmeyer & Öğmen, 2006), using a thresholding procedure to find each participant's threshold. Finding each participant's threshold allows for obtaining a good mix of stimuli giving rise to different levels of subjective experience. We focused on the early visual activity (VAN) and the late frontal activity (LP) as many EEG and MEG studies have done (for a comprehensive review of studies, see Förster et al., 2020). Thus, we are here not looking into the neural correlates of consciousness of states, such as coma and dreamless sleep, but the neural correlates of the consciousness of the specific content of visual stimuli.

**FIGURE 1:**
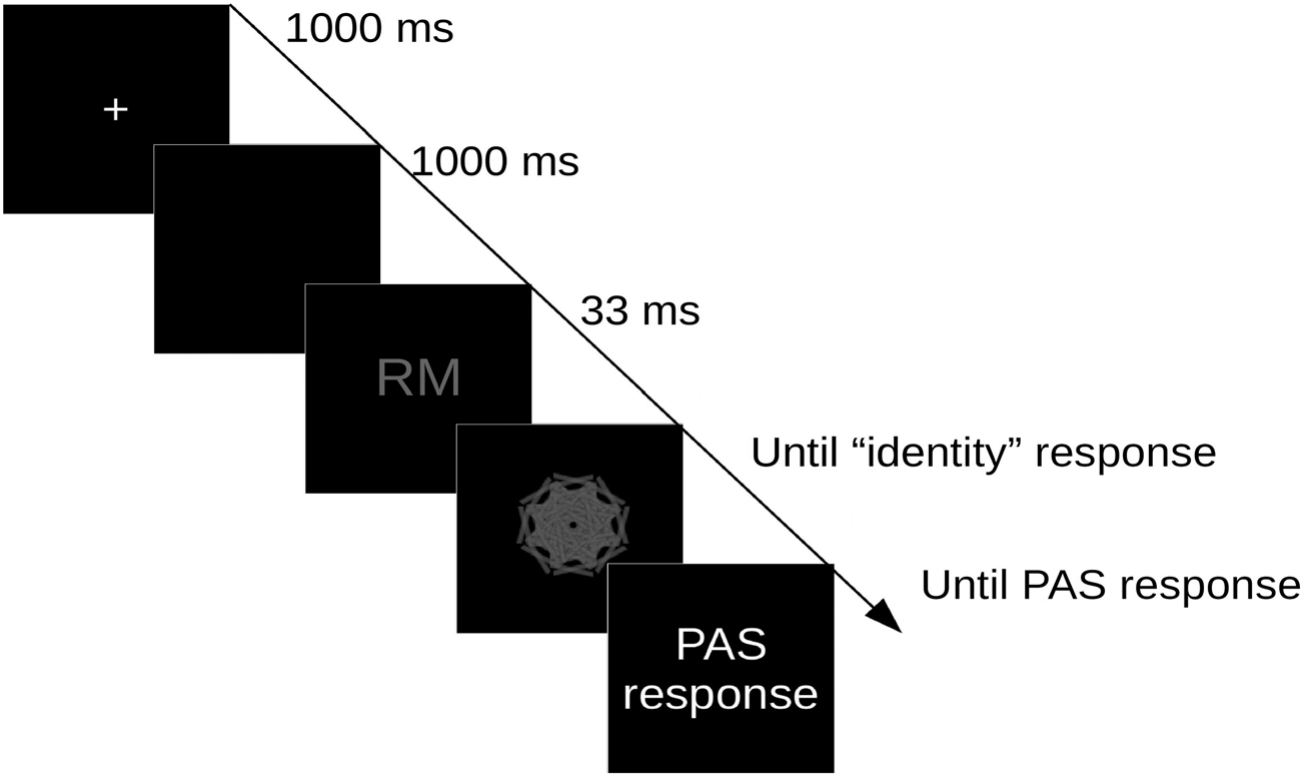
Paradigm: A fixation cross was presented for 1000 ms, followed by a delay of 1000 ms, to prevent forward masking of the target stimulus. A pair of letters was then presented for 33 ms immediately followed by a mask that remained on until the participant indicated whether the two letters were ‘same’ or ‘different’. In the perceptual task, the target letters were defined as ‘same’ if they were identical, for example, ‘RR’, and ‘different’ in all other cases. For the conceptual task, the target letters were defined as ‘same’ if they were of the same type according to whether they were consonants or vowels, for example, ‘EU’ or ‘SV’, and ‘different’ if they were of opposite types, for example, ‘EV’. After that, participants had to indicate perceptual experience by one of four ratings, no experience, weak glimpse, almost clear experience or clear experience.

We used the classification tasks of Posner and Mitchell (1967), as these tasks allow for different levels of processing despite identical stimuli. Specifically, in the *perceptual* task, participants had to indicate whether or not two letters were identical, whereas in the *conceptual* task, they had to indicate whether or not two letters were both vowels and consonants (Figure 1). This approach of task demands may be seen within the levels of processing hypothesis presented by Windey and Cleeremans (2015), where they hypothesize that low‐level visual experience is graded, such as perception of colour, and that high‐level visual experience, such as perception of animacy, is categorical. In fact, Jimenez et al. (2021) find that in high‐level visual experience, the LP is more strongly modulated than in low‐level visual experience. From a more general viewpoint, the current study may also inform the debate about whether the global neuronal workspace theory or the integrated information theory fits the experimental results the best (Melloni et al., 2021), while not being able to adjudicate finally between them.

We used the Perceptual Awareness Scale (PAS) (Ramsøy & Overgaard, 2004) to let participants rate their subjective experiences of the presented stimuli. PAS has four rating points, no experience (NE), weak glimpse (WG), almost clear experience (ACE) and clear experience (CE) (Table 1). The differences between neighbouring points are as follows: *there being a conscious experience at all* (WG vs. NE), *there being a conscious experience of content* (ACE vs. WG) and *there being a conscious experience of unambiguousness of the content* (CE vs. ACE).

**TABLE 1:** The Perceptual Awareness Scale (PAS)

Label	Description (from Ramsøy & Overgaard, 2004)
(1) No experience (NE)	No impression of the stimulus. All answers are seen as mere guesses
(2) Weak glimpse (WG)	A feeling that something has been shown. Not characterized by any content, and this cannot be specified any further
(3) Almost clear experience (ACE)	Ambiguous experience of the stimulus. Some stimulus aspects are experienced more vividly than others. A feeling of almost being certain about one's answer
(4) Clear experience (CE)	Non‐ambiguous experience of the stimulus. No doubt in one's answer

*Note*: Scale steps and their descriptions.

We hypothesized that the neural generators of the VAN and LP would depend on the subjective experience, as measured by PAS, and that this in turn would depend on the task of the participant, *perceptual* or *conceptual* (Figure 1). More specifically, we expected that the *perceptual* task would recruit more early visual activity, as the clarity of subjective experience increased, than the *conceptual* task, and that, vice versa, the *conceptual* task would recruit more late frontal activity than the *perceptual* task, as the clarity of subjective experience increased.

Using PAS is pertinent to showing that task requirements may affect neural correlates of consciousness, as both the VAN and the LP correlate with the level of experience reported using PAS (Andersen et al., 2016; Tagliabue et al., 2016). We can then investigate how differences in neural activity manifest between adjacent ratings of PAS when task requirements differ.

Specifically, based on the idea that the LP is more strongly modulated by high‐level visual experience (Jimenez et al., 2021) (our conceptual task) and that the VAN is more strongly modulated by low‐level visual experience (our perceptual task), we hypothesized that the neural generators of the VAN and the LP would depend on the subjective experience, as measured by the PAS, and that this in turn would depend on the task of the participant, perceptual or conceptual (Figure 1). More specifically, we expected that the perceptual task would recruit more early visual activity, as the clarity of subjective experience increased, than the conceptual task, and that, vice versa, the conceptual task would recruit more late frontal activity than the perceptual task, as the clarity of subjective experience increased. In statistical terms, we should thus expect an interaction between task (conceptual), brain area (visual or frontal), time range (early or late) and subjective experience (NE, WG, ACE or CE).

## 2 METHODS

### 2.1 Participants

Forty right‐handed participants, 18 women and 22 men, with normal or corrected‐to‐normal vision able to read Danish, which uses the Latin alphabet, signed up to participate in the study. All provided written informed consent prior to their participation. The median age was 23 years (range: 20 to 31 years). The experiment was approved by the local ethics committee, De Videnskabsetiske Komitéer for Region Midtjylland, and carried out in accordance with the Declaration of Helsinki. One participant was excluded from the study before the magnetoencephalographic recording, due to having a metallic wire from dental braces behind his teeth. One participant did not show up for the magnetic resonance imaging session. Data from one participant were rejected because the cortical surfaces could not be reconstructed with freesurfer. Finally, one participant had all his trials removed by the rejection threshold applied below, and this was not restricted to a single or few channels. This left 36 participants, 19 having completed the perceptual task and 17 having completed the conceptual task.

### 2.2 Stimuli and procedure

Participants were seated 137 cm from a screen onto which a Panasonic PT‐D10000E projector projected an image with a resolution of 1280 × 800 pixels and a refresh rate of 60 Hz. A fixation cross was presented for 1000 ms followed by a delay (a blank screen) of 1000 ms (to prevent forward masking), which was followed by the target, a pair of letters, presented for 33.3 ms. A mask was presented after the target and remained until the participant had responded. The participants were instructed to respond to the stimuli as fast and as accurately as possible according to the specifications described below (Figure 1).

Participants were administered one of two tasks where they had to judge whether two letters presented simultaneously were the same or different according to the specific instruction. In the *perceptual* task, the target was defined as ‘same’ if the letters were identical, for example, ‘RR’, and ‘different’ in all other cases. For the *conceptual* task, the target was defined as ‘same’ if the letters were of the same type in terms of consonanthood and vowelhood, for example, the same for ‘EU’ or ‘SV’, and different if the letters were of different types, for example, ‘EV’. After performing the same/different judgement, participants were to report their conscious experience of the target using the PAS (Ramsøy & Overgaard, 2004; see Table 1). All stimuli were presented using psychopy (Peirce et al., 2019). The experiment consisted of 800 trials presented in blocks of 100 trials each; 10 trials in each block were catch trials where no stimulus was shown during the target presentation. In between blocks, participants were allowed a short break if needed. After each block, the response hand for the same/different judgement changed.

Twenty of the participants performed the *perceptual* task, and the remaining twenty performed the *conceptual* task. The task administered to a participant was determined by having odd‐numbered participants perform the perceptual task and even‐numbered participants perform the conceptual task. The study was done as a mixed‐factorial design with the task variable being a between‐participants variable due to the worry that it would not be possible to control the order effect. Specifically, if a participant would have run the conceptual task before the perceptual task, it is possible that consonanthood and vowelhood representations would be activated again.

The number of participants and trials were chosen based on Andersen et al. (2016), as this study elicited both the VAN and the LP.

The background was black (RGB values: 0, 0 and 0). The fixation cross was white (RGB values: 255, 255 and 255), and the height and width of this cross were 0.56° of visual angle. The thickness of the bars was 0.05° of visual angle. The luminance of the target letters was determined by using a thresholding procedure as described below. The height of the letter was 0.75° of visual angle and presented with a monospaced font (https://www.gnome-look.org/p/998559 [date last accessed: 13 September 2022]). The mask was a grey hue (RGB values: 122.5, 122.5 and 122.5).

Before participants were prepared for the magnetoencephalographic recording (see below), they completed a practice session to ascertain that they understood the task assigned. This session consisted of 21 trials with targets of varying contrast and was done in the magnetically shielded room where the recording was made.

After the preparation for the magnetoencephalographic recording had been finished, participants completed a thresholding procedure for finding a contrast threshold value that would be used throughout the remainder of the experiment. MEG was also recorded during the thresholding procedure.

### 2.3 Thresholding procedure

The target letters were presented in greyscale. Participants started at the grey hue (RGB values: 122.5, 122.5 and 122.5). We used a stochastic approximation staircase to change the grey hue after each trial (Faes et al., 2007), aiming at a proportion correct of 0.75. The contrast was held constant after the thresholding procedure throughout the remainder of the experiment. This threshold was chosen in order to ensure that ample data were obtained for both correct and incorrect responses as well as for each PAS rating. Frequently, studies use staircases with a slightly lower desired accuracy (around 70%), but because PAS ratings increase relatively slower than accuracy (Sandberg et al., 2011), we opted for a slightly higher accuracy of 75% while avoiding higher accuracies to minimize the risk of ceiling performance. The contrast was held constant after the thresholding procedure throughout the remainder of the experiment. The median contrast of the final letters presented across participants was RGB values (33.5, 33.5 and 33.5). The 25th quantile was RGB values (26.2, 26.2 and 26.2). The 75th quantile was RGB values (62.8, 62.8 and 62.8). The median number of trials before the staircase converged was 126.

### 2.4 MEG recordings

Before the magnetoencephalographic recording, we fastened four head position indicator coils on the participants, one behind each ear and one on the left and right temples, respectively. Three fiducial points, the nasion and the left and right pre‐auricular points, were digitized along with the positions of the four coils. Furthermore, around 200 extra points, digitizing the head shape of the participant, were acquired. The digitization was done using a Polhemus FASTRAK Digitizer (Colchester, VT, USA). A head coordinate system was created based on the fiducials, and this coordinate system was later co‐registered to anatomical magnetic resonance images to enable the estimation of individual forward models, linking each participant's source model to the sensor array.

Magnetoencephalographic data were recorded in a magnetically shielded room with an Elekta Neuromag TRIUX system with 102 magnetometers and 204 planar gradiometers with a sampling frequency of 1000 Hz and an online high‐pass filter of 0.1 Hz and a low‐pass filter of 330 Hz.

Subsequently, we analysed the data offline using mne‐python (Gramfort et al., 2013). We appplied a low‐pass filter of 40 Hz (one‐pass, non‐causal; finite impulse response; zero‐phase; upper transition bandwidth: 10.00 (−6 dB cut‐off frequency: 45 Hz, filter length: 331 samples; and 0.0194 passband ripple; and 53 dB stopband attenuation), and then cut the raw data into epochs of 1000 ms, 200 ms pre‐stimulus and 800 ms post‐stimulus. Epochs were demeaned using the mean value of the pre‐stimulus period. Epochs were downsampled to a sampling frequency of 250 Hz. Epochs of data including magnetometer responses greater than 4 pT or gradiometer responses of 400 pT/cm or electro‐oculographic response greater than 250 μV were rejected. If the inclusion of a single channel resulted in more than 100 epochs having to be removed that channel was removed instead. On average, 334 trials were left per subject after rejection of noisy trials. On average, 333 of the removed trials were removed due to eye blinks. This is a high proportion, but as the hypothesis of the study was that frontal activity would increase in the conceptual task, we chose a conservative approach, such that ocular activity would not confound the interpretation of frontal activity. The mean number of trials left in the perceptual group was 394, and in the conceptual group, it was 267 trials. The null hypothesis that the number of trials was the same in the two groups could not be rejected, *t*_34_ = 1.54, *p* = 0.13. The epochs were categorized according to the four rating steps of the PAS. Eight signal space projectors estimated from empty room recordings were projected out from each participant's recording.

### 2.5 Source reconstruction

Source reconstruction was done using the sLORETA algorithm (Pascual‐Marqui, 2002) based on the inverse imaging implementations from mne‐c (Gramfort et al., 2014). Source reconstructions were done for each participant based on participant‐specific cortical reconstructions and volumetric segmentations done using freesurfer (Dale et al., 1999; Fischl et al., 1999). These segmentations were based on T1 images obtained from a magnetic resonance imaging sequence from a Siemens MAGNETOM Trio. The pulse sequence parameters were as follows: 1 mm isotropic resolution; field of view: 256 × 256 mm; 176 slices; slice thickness: 1 mm; bandwidth: 150 Hz/pixel; flip angle: 9°; inversion time (TI): 960 ms; echo time (TE): 3.70 ms; and repetition time (TR): 2420 ms. Subsequently, we delineated the head and brain surfaces using the watershed algorithm from freesurfer. We created a cortical source space with around 8000 sources placed equidistantly along the pial surface.

Single‐compartment boundary element method solutions were estimated from the inner skull boundary for each participant. Based on the boundary element method solutions, the cortical source space, the information about the sensor positions and the co‐registration between the sensor space and the T1 image, forward models were created for each participant. Source reconstruction was done on the evoked response based on all the trials, collapsed across PAS ratings, for each participant. This was used as a localizer to inform the exact occipital and frontal areas and the boundaries of the time range for the early and late ranges, respectively. The source reconstruction procedure was then applied to each trial using the sLORETA algorithm and categorized according to its PAS rating. The single‐trial source reconstructions were regularized with a regularization value three times greater than the reconstructions based on evoked responses, which were done using the standard settings of mne‐python. This secured a smoother solution of the otherwise more noisy epochs (compared to evoked responses). In all cases, pre‐whitening was applied, allowing the use of both magnetometers and gradiometers, based on estimating the noise covariance using the epoch activity from −200 to 0 ms. The single‐trial reconstructions were used in the statistical analysis.

Regions of interest were defined by averaging trials across all PAS ratings for each participant and subsequently doing a grand average across participants irrespective of task. We used the *Desikan–Killiany* atlas (Desikan et al., 2006) as implemented in freesurfer to find the regions that showed the greatest activity in the collapsed grand averages and used these as the regions of interest in the statistical analysis. In the same way, the early and late time ranges were defined from the maximal responses in the collapsed grand averages. From each participant, we only included responses from a given PAS rating if the given participant had at least 20 instances of it (Figure 4a). This was a good compromise between having enough trials to estimate a likely mean response for each participant and not rejecting too many participants. For the dependent variable, the mean amplitude over each time range and region of interest for each trial according to its PAS rating was calculated.

### 2.6 Statistical analyses

For both the analysis of the behavioural data and the analysis of the magnetoencephalographic data, mixed‐effects models (McCulloch & Neuhaus, 2005) were fitted based on a combination of fixed and random effects. For the behavioural data analyses, we performed model comparisons between models that did or did not include the relevant effects and interactions to find the best compromise between an explanatory and a parsimonious model. This was done using the log‐likelihood ratio between two given models, as this ratio is approximated by a *χ* ^2^ distribution. Whether the null hypothesis, that the log‐likelihood ratio between the two models is equal to zero, can be rejected can be tested using a *χ* ^2^ test with the log likelihood being the test statistic and the degrees of freedom being equal to the difference in free parameters of the two models. We rejected the null hypothesis at *α* = 0.05.

Mixed‐effects models were chosen due to the nature of the present data where it is impossible to fully control that participants rate an equal number of trials into each of the four categories of the PAS. Mixed‐effects models handle unbalanced designs well, because the contribution of each participant to the group average is weighted based on the number of trials. They are also more powerful than conventional designs, where only fixed effects are tested after each participant has had their responses reduced to a summary statistic (e.g., the mean). This is because the within‐participant variance is modelled, instead of discarded, as is the case when each participant is reduced to a summary statistic.

The mixed models were estimated using the *lme4* package (Bates et al., 2014) from r (R Core Team, 2020).

#### 2.6.1 Response times and task accuracy

For the behavioural data, the dependent variables of interest were response times and accuracy. Response times were log transformed and modelled as a normal parameter, and task accuracy (correct/wrong answer) was modelled as a binomial parameter using single‐trial response times. Otherwise, the same model was fitted as above. These were modelled as being dependent on PAS rating (within‐group: four levels: NE, WG, ACE and CE), task (between‐group: two levels: perceptual and conceptual) and the interaction between them. For random effects, individual intercepts were modelled for each participant (36). We tested the differences between PAS rating between the two tasks, using a Wald test.

Note that for both analyses, all trials are included, and all participants who finished the behavioural task are included.

#### 2.6.2 Neural responses—MEG

For the magnetoencephalographic data, four independent variables were defined: PAS rating (within‐group: four levels: NE, WG, ACE and CE), task (between‐group: two levels: perceptual and conceptual), time range (within‐group: two levels: early and late) and region of interest (within‐group: two levels: occipital and frontal), resulting in a mixed‐factorial design. For random effects, individual intercepts were modelled for each participant (36).

We expected a four‐way interaction between the four variables tested, given the hypothesis that task and strategy influence the neural correlates of consciousness, as the task and strategy implemented (task) would recruit resources differently dependent on temporal extent of processing (time range), brain areas included (region of interest) and the level of perceptual experience (PAS rating).

Four‐way interactions are hard to interpret, so within this model, we were interested in six specific differences, namely, whether the differences between the neighbouring PAS ratings would differ between the two tasks. For this purpose, we tested six null hypotheses. Note that testing the differences of differences means that potential individual differences in the offset of the underlying brain response will be subtracted out.

Early range (in the occipital lobe):

1. H_0_: (WG_CONCEPTUAL_ − NE_CONCEPTUAL_) − (WG_PERCEPTUAL_ − NE_PERCEPTUAL_) = 0
2. H_0_: (ACE_CONCEPTUAL_ − WG_CONCEPTUAL_) − (ACE_PERCEPTUAL_ − WG_PERCEPTUAL_) = 0
3. H_0_: (CE_CONCEPTUAL_ − ACE_CONCEPTUAL_) − (CE_CONCEPTUAL_ − ACE_CONCEPTUAL_) = 0

Late range (in the frontal lobe):

- H_0_: (WG_CONCEPTUAL_ − NE_CONCEPTUAL_) − (WG_PERCEPTUAL_ − NE_PERCEPTUAL_) = 0
- H_0_: (ACE_CONCEPTUAL_ − WG_CONCEPTUAL_) − (ACE_PERCEPTUAL_ − WG_PERCEPTUAL_) = 0
- H_0_: (CE_CONCEPTUAL_ − ACE_CONCEPTUAL_) − (CE_CONCEPTUAL_ − ACE_CONCEPTUAL_) = 0

For testing these null hypotheses, the Wald test was used using the *linearHypothesis* function, as implemented in the *car* package (Fox & Weisberg, 2019) from r. The Wald test statistic follows an asymptotic *χ* ^2^ distribution with degrees of freedom equal to the number of tests made. We rejected the null hypothesis at *α* = 0.05.

## 3 RESULTS

### 3.1 Behavioural performance

**FIGURE 2:**
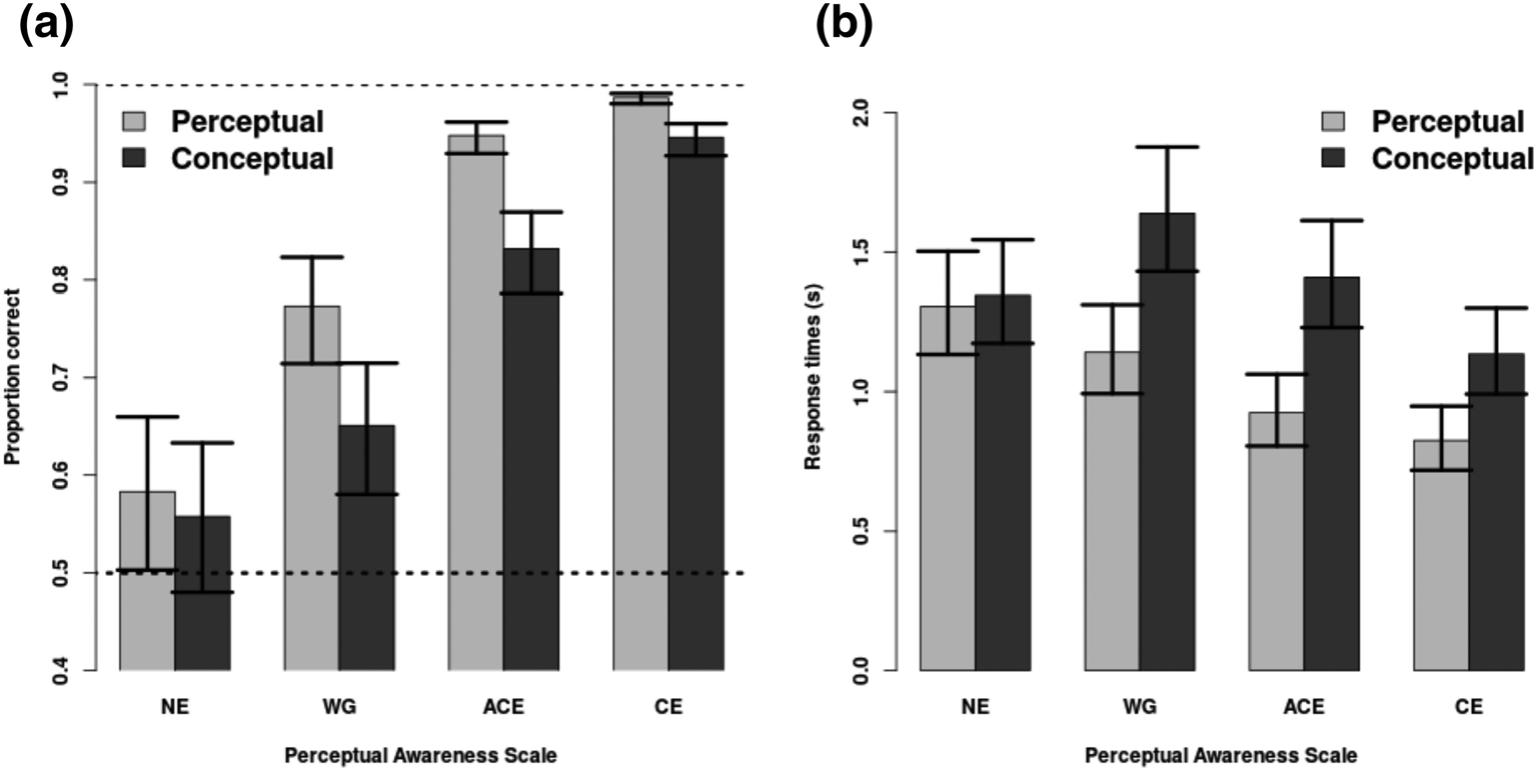
Behavioural performance. (a) Accuracy for each of the PAS ratings per task. Error bars are 95% confidence intervals. (b) Response times for each of the PAS ratings per task. Error bars are 95% confidence intervals.

For the proportion of correct responses, the interaction between PAS rating and task could not be dropped without a significant change in log likelihood, *χ* ^2^(3) = 93.5, *p* < 0.001. This interaction was driven by the proportion of correct responses being lower for WG, ACE and CE in the conceptual task compared to the perceptual task (Figure 2a), as was shown by the fact that the null hypothesis that the proportion of correct responses was the same between tasks could be rejected for these three ratings: WG: *χ* ^2^(1) = 6.07, *p* = 0.0138; ACE: *χ* ^2^(1) = 29.7, *p* < 0.001; CE: *χ* ^2^(1) = 24.2, *p* < 0.001; but not for NE: *χ* ^2^(1) = 0.322, *p* = 0.570. For both the conceptual task and the perceptual task, accuracy was higher for the higher awareness rating when comparing neighbouring ratings, that is, WG versus NE, ACE versus WG, and CE versus ACE (all [six] *χ* ^2^(1)s > 37.7; all *p*s < 0.001).

For response times, the interaction between PAS rating and task could not be dropped without a significant change in log likelihood, *χ* ^2^(3) = 324, *p* < 0.001. This interaction was driven by slower response times for WG, ACE and CE in the conceptual task compared to the perceptual task (Figure 2b), as is shown by the fact that the null hypothesis that proportion of correct responses was the same between tasks could be rejected for these three ratings: WG: *χ* ^2^(1) = 10.5, *p* = 0.00118; ACE: *χ* ^2^(1) = 15.9, *p* < 0.001; CE *χ* ^2^(1) = 6.43, *p* = 0.012; but not for NE: *χ* ^2^(1) = 0.231, *p* = 0.630.

### 3.2 Catch trials

The median number of times participants used the four different PAS ratings on the catch trials for the perceptual task was as follows: NE: 71; WG: 8; ACE: 0; and CE: 0; and for the conceptual task: NE: 74; WG: 2; ACE: 0; and CE: 0. This overall indicates that participants were capable of teasing apart veridical stimuli from catch trials.

### 3.3 MEG

#### 3.3.1 Collapsed analysis

**FIGURE 3:**
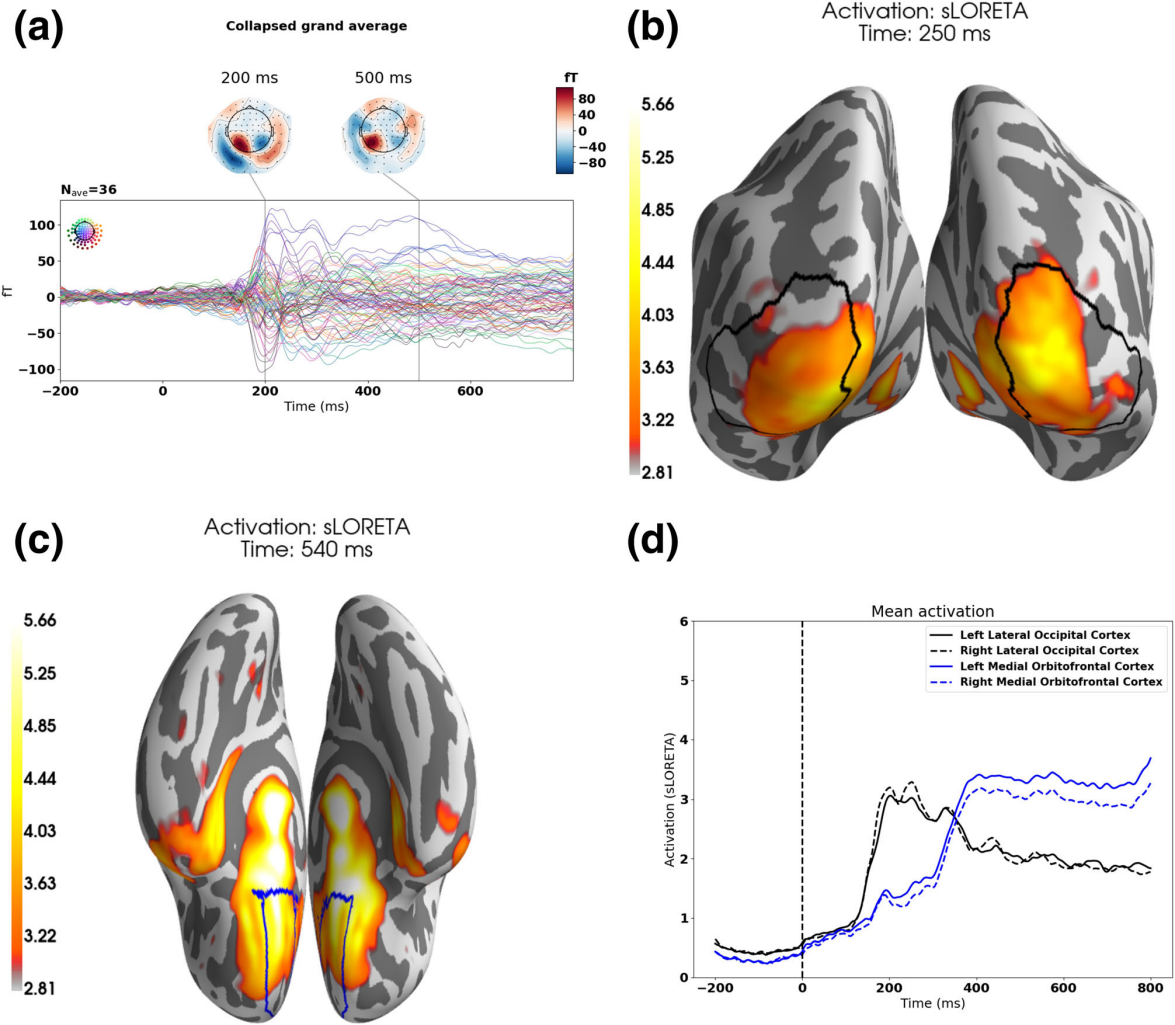
Collapsed activations—grand average. (a) Grand average over sensors for the collapsed activation. (b) An early activation (250 ms) is seen over the lateral occipital cortex and (c) a late activation (540 ms) over the medial orbitofrontal cortex. (d) Time courses for the indicated regions. The mean over all sources within the region was found for each time point. On the participant level, trials were averaged across all PAS ratings, and subsequently, a grand average across participants was done irrespective of task. The lateral occipital cortex and the medial orbitofrontal cortex were defined using the Desikan–Killiany atlas as implemented in freesurfer (Desikan et al., 2006).

Based on the source analysis of evoked responses collapsed over PAS ratings and over each task (Figure 3), we identified two regions of interest, the lateral occipital lobe and the medial orbitofrontal lobe. We identified two time ranges, from 100 to 400 ms for the early visual response and from 400 to 700 ms for the late frontal response.

#### 3.3.2 Modelling the responses

For the magnetoencephalographic responses, the four‐way interaction between PAS rating, task, time range and region of interest could not be dropped without a significant change in log likelihood, *χ* ^2^(3) = 12.4, *p* = 0.00607.

#### 3.3.3 Testing the null hypotheses

Wald tests were done for each of the six null hypotheses (Table 2).

**TABLE 2:** Statistics for the a priori comparisons

Null hypothesis (H_0_)	Test statistic	*p* value	H_0_ rejected/not rejected
Early range (100–400 ms) (in the occipital lobe)			
(WG_CONCEPTUAL_ − NE_CONCEPTUAL_) − WG_PERCEPTUAL_ − NE_PERCEPTUAL_) = 0	*χ* ^2^(1) = 6.05	0.0139	Rejected
(ACE_CONCEPTUAL_ − WG_CONCEPTUAL_) − (ACE_PERCEPTUAL_ − WG_PERCEPTUAL_) = 0	*χ* ^2^(1) = 2.63	0.105	Not rejected
(CE_CONCEPTUAL_ − ACE_CONCEPTUAL_) − (CE_CONCEPTUAL_ − ACE_CONCEPTUAL_) = 0	*χ* ^2^(1) = 5.55	0.0184	Rejected
Late range (400–700 ms) (in the frontal lobe)			
(WG_CONCEPTUAL_ − NE_CONCEPTUAL_) − (WG_PERCEPTUAL_ − NE_PERCEPTUAL_) = 0	*χ* ^2^(1) = 13.7	<0.001	Rejected
(ACE_CONCEPTUAL_ − WG_CONCEPTUAL_) − (ACE_PERCEPTUAL_ − WG_PERCEPTUAL_) = 0	*χ* ^2^(1) = 0.193	0.660	Not rejected
(CE_CONCEPTUAL_ − ACE_CONCEPTUAL_) − (CE_CONCEPTUAL_ − ACE_CONCEPTUAL_) = 0	*χ* ^2^(1) = 5.59	0.0181	Rejected

#### 3.3.4 The modelled responses

**FIGURE 4:**
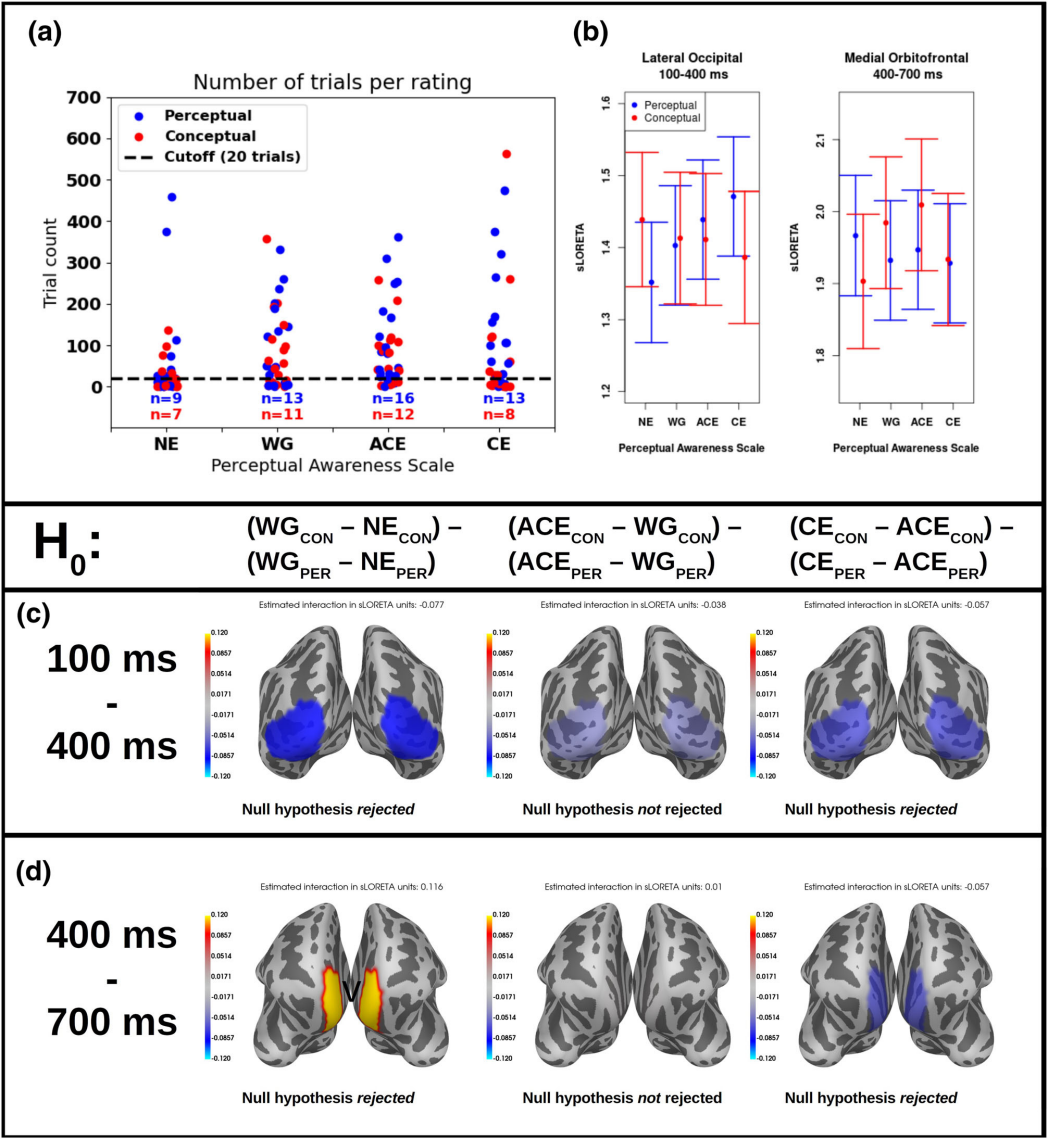
Estimated main effects and tested interactions. (a) Number of trials per participant and how many participant data sets survived the cut‐off (minimum 20 trials) per PAS rating. (b) Estimated means of activity in the two labels and their associated standard errors. (c) The estimated interaction effect for the lateral occipital area over the early range (100–400 ms). (d) The estimated interaction effect for the medial orbitofrontal cortex over the late range (400–700 ms)

For descriptive purposes, we show the mean of the estimated mean responses in the two regions of interest (Figure 4b). For the lateral occipital area, we found an increase of mean activity as experiences became clearer in the perceptual task, but a decrease in the conceptual task. For the medial orbitofrontal cortex, we found a more variable pattern. The estimated interaction effects on the associated regions of interest can be seen in Figure 4c,d.

## 4 DISCUSSION

The behavioural results showed that the clearer the experiences of participants, the better their performance was, in terms of both accuracy and response times (Figure 2). Differences were found between the perceptual and conceptual tasks for both accuracy and response time. However, no differences were found between tasks for NE for neither response times nor accuracy. We can thus conclude that the perceptual task was easier than the conceptual task and that this was driven by accuracy being better and responses being faster on ratings WG and above. The possible confounding effect of this difference of the magnetoencephalographic data would mostly be expressed in the offset of the magnetoencephalographic response—this potential difference in offset is counteracted by focusing on interactions instead of main effects (Table 2).

For the magnetoencephalographic data, we found the expected early visual and late frontal responses (Figure 3). (Similar frontal responses have also been found for the auditory domain [Eklund et al., 2019].) We furthermore found that the task of the participant influenced the neural activity related to subjective experiences in the visual and frontal areas. Overall, our hypothesis that the perceptual task would recruit more early visual activity than the conceptual task as the clarity of the subjective experience increased was corroborated by the data (Table 1 and Figure 4b,c). The hypothesis that vice versa the conceptual task would recruit more late frontal activity than the perceptual task as the clarity of subjective experience increased was only corroborated for the comparison between WG and NE. However, for the comparison between CE and ACE, we, opposite to the hypothesis, found that the difference in the perceptual task for the late frontal activity was greater than the difference for the conceptual task. However, for the comparison between CE and ACE, we found—contrary to our hypotheses—that the (signed) difference between CE and ACE in the perceptual task for the late frontal activity was greater than the (signed) difference between CE and ACE for the conceptual task (Figure 4d, right panel). This finding suggests that the difference between the two conditions is not simply a confound based on one condition involving relatively more attention or working memory load, and it could be interpreted to suggest that the two conditions involve different cognitive or neural strategies (e.g., Mogensen & Overgaard, 2020), or, in other words, that the two different instructions for the same task lead to the recruitment of different neural processes and thus involve a difference in what can be considered the neural correlates of consciousness. In fact, it may be clearest in the differences, between the two tasks, between WG and NE. As no information is likely to be available for executing the task when NE is reported (Figure 2a), the difference in differences across tasks between WG and NE is likely to reflect the difference in frontal activation between the two tasks. Following this interpretation, our results indicate that the execution of the conceptual task recruits more frontal activity than the execution of the perceptual task. An alternative interpretation of the finding that CE did not result in greater frontal activation than ACE is that the LP is simply not a good candidate of a neural correlate of consciousness, but as the LP has been found to scale linearly with the level of experience in several other studies (Andersen et al., 2016; Del Cul et al., 2007; Tagliabue et al., 2016), it is likely that the differences reported here are due to our task manipulation. However, it has also been argued that the LP unfolds as an all‐or‐none response (Dehaene, 2014; Sergent et al., 2005) that may not be able to differentiate between ACE and CE.

An alternative view is that the frontal activity is part of the neural correlates of consciousness, as the task, requiring categorization into vowels and consonants, shapes the perception or, in other words, that perceptual experience differs substantially between conditions. This could be taken to suggest that phenomenal consciousness and access consciousness are fundamentally linked—a view that has been defended several times (e.g., Kouider et al., 2012; Mogensen & Overgaard, 2020; Schlicht, 2012).

The present data thus support a view where the task at hand influences the neural correlates of consciousness; that is, despite identical stimuli and thus similar subjective experiences that share many properties, there are still differences in how the stimuli are processed in visual cortex. One possibility is that participants in the perceptual task are attending more to the shapes and edges of the presented letters, that is, to be able to ascertain whether the letters are identical, whereas in the conceptual task, attention is focused on extracting which letter it is, for which it may be sufficient to extract just the information that allows for classifying it as a given letter. In this case, we may see attention as a confounding factor, leaving it unclear what the *correct* minimal conditions sufficient for realizing a conscious representation are—is it the processing of edges and shapes in general, or is it the processing of edges and shapes sufficient for inferring the letter presented? In this sense, what attention is directed to may be part of the strategy applied by the subject.

The finding of more frontal activity in the perceptual task for CE compared to ACE was not initially expected, but does not pose an insurmountable challenge to the view that neural correlates of consciousness depend on the task at hand. One explanation may be that when information is fully subjectively available (as they are assumed to be in CE), more information may be accessed than is strictly necessary for the perceptual task; for example, one may automatically categorize the letters as, say two As, as information for the report, whereas with less than CEs, one may be more relying on the comparison of shapes. In contrast to this, the information about what letter was presented must have been retrieved already for WG and ACE (as above‐chance performance shows [Figure 2a] for the conceptual task and more so than for the perceptual task). Thus, the difference in frontal activity between CE and ACE may in these conditions thus be greater in perceptual tasks than in conceptual tasks, as the data here indicate. This suggests that the strategies that participants use may not be fully orthogonal on another when stimuli are clearly perceived, but more so when they are less clearly perceived.

These examples are just speculations of how one may imagine that participants apply different task solution strategies related to the instructions. Whereas they may be true or false and should be investigated further, the results at hand suggest that the neural correlates of consciousness may not just be confounded by but actually depend on the task.

## 5 CONCLUSION

The current study provides evidence that even when stimuli are identical, the neural activity related to the subjective experience of these stimuli depends on the task at hand. This is evidence that an appropriate framework for the search for the neural correlates of consciousness is a framework that allows for subjective experience to realize differently according to the task at hand and that there is likely not just one neural correlate of consciousness.

## CONFLICTS OF INTEREST

The authors declare no conflicts of interest.

## AUTHOR CONTRIBUTIONS

L. M. Andersen designed the study, collected and analysed the data, and drafted and wrote the final version of the paper. M. C. Vinding collected the data, provided critical feedback to the draft and took part in finalizing the paper. K. Sandberg and M. Overgaard designed the study, provided critical feedback to the draft and took part in finalizing the paper.

### PEER REVIEW

The peer review history for this article is available at https://publons.com/publon/10.1111/ejn.15820.

## Data Availability

Data are available on request due to privacy/ethical restrictions. Data cannot be made publicly available as we do not have permission to share data publicly.
